# *Cinnamomum cassia* Essential Oil Inhibits α-MSH-Induced Melanin Production and Oxidative Stress in Murine B16 Melanoma Cells

**DOI:** 10.3390/ijms140919186

**Published:** 2013-09-18

**Authors:** Su-Tze Chou, Wen-Lun Chang, Chen-Tien Chang, Shih-Lan Hsu, Yu-Che Lin, Ying Shih

**Affiliations:** 1 Department of Food and Nutrition, Providence University, 200, Sec. 7, Taiwan Boulevard, Shalu Dist., Taichung City 43301, Taiwan; E-Mails: stchou@pu.edu.tw (S.-T.C.); ctchang@pu.edu.tw (C.-T.C.); 2 Department of Cosmetic Science, Providence University, 200, Sec. 7, Taiwan Boulevard, Shalu Dist., Taichung City 43301, Taiwan; E-Mails: g9924002@gmail.com (W.-L.C.); g1000208@pu.edu.tw (Y.-C.L.); 3 Department of Applied Chemistry, Providence University, 200, Sec. 7, Taiwan Boulevard, Shalu Dist., Taichung City 43301, Taiwan; 4 Department of Education and Research, Taichung Veterans General Hospital, 1650, Sec. 4, Taiwan Boulevard, Taichung City 40705, Taiwan; E-Mail: h2326@vghtc.gov.tw

**Keywords:** *Cinnamomum cassia* Presl, essential oil, cinnamaldehyde, tyrosinase, melanin, antioxidant

## Abstract

Essential oils extracted from aromatic plants exhibit important biological activities and have become increasingly important for the development of aromatherapy for complementary and alternative medicine. The essential oil extracted from *Cinnamomum cassia* Presl (CC-EO) has various functional properties; however, little information is available regarding its anti-tyrosinase and anti-melanogenic activities. In this study, 16 compounds in the CC-EO have been identified; the major components of this oil are *cis*-2-methoxycinnamic acid (43.06%) and cinnamaldehyde (42.37%). CC-EO and cinnamaldehyde exhibited anti-tyrosinase activities; however, *cis*-2-methoxycinnamic acid did not demonstrate tyrosinase inhibitory activity. In murine B16 melanoma cells stimulated with α-melanocyte-stimulating hormone (α-MSH), CC-EO and cinnamaldehyde not only reduced the melanin content and tyrosinase activity of the cells but also down-regulated tyrosinase expression without exhibiting cytotoxicity. Moreover, CC-EO and cinnamaldehyde decreased thiobarbituric acid-reactive substance (TBARS) levels and restored glutathione (GSH) and catalase activity in the α-MSH-stimulated B16 cells. These results demonstrate that CC-EO and its major component, cinnamaldehyde, possess potent anti-tyrosinase and anti-melanogenic activities that are coupled with antioxidant properties. Therefore, CC-EO may be a good source of skin-whitening agents and may have potential as an antioxidant in the future development of complementary and alternative medicine-based aromatherapy.

## 1. Introduction

Melanogenesis occurs in melanocytes through an enzymatic process that is catalyzed by tyrosinase. Tyrosinase (monophenol, dihydroxyphenylalanine: oxygen oxidoreductase, EC 1.14.18.1) is a multifunctional, copper-containing enzyme that is widely distributed in nature and plays a key role in melanogenesis. In particular, it catalyzes both the hydroxylation of l-tyrosine (monophenolase activity) and the oxidation of l-DOPA (diphenolase activity) to *o*-quinone, which induces the production of melanin pigments [1]. The tyrosinase catalysis is regulated by α-melanocyte-stimulating hormone (α-MSH), which is known to be a melanotropic hormone and a major physiologic stimulus for murine pigmentation [2]. Melanin production is considered to be responsible for skin color and plays a significant role in photoprotection; however, the overproduction and excessive accumulation of melanin leads to various human skin disorders, such as melasma, freckles, age spots, and malignant melanomas [3]. Therefore, the increasing desire of skin whitening agents from synthetic [4,5] or natural [6,7] resources for both beauty and therapeutic purposes is under development. Plant-derived compounds including gallic acid [8], vanillic acid [9], eugenol [10], and the recently characterized substance of curcumin [11] have been reported to possess anti-melanogenic abilities.

*Cinnamomum cassia* Presl is widely cultivated in China. The dried stem bark of *C. cassia*, *i.e.*, cassia bark, is not only important as a food spice, but is also considered to have medicinal properties, such as antimicrobial [12], antitumorigenic [13], anti-inflammatory [14], and antidiabetic characteristics [15]. In addition, the methanol extract of *C. cassia* twigs was found to possess tyrosinase inhibitory activity [16]. It is known that plant essential oils (EOs) can have various functional properties, such as a pleasant aroma; the ability to repel various animals, including insects; and the capacity to inhibit microorganisms. Moreover, various products made from EOs have been used in aromatherapy and may alleviate or stabilize certain physical and psychological conditions [17]. The anise and citrus essential oils have been reported to possess tyrosinase inhibitory activities [17,18]. The essential oil from the cinnamon species *C. zeylanicum* Blume has been reported to show anti-tyrosinase activity, and cinnamaldehyde was found to be the primary compound responsible for this inhibition [19]. The essential oil that is extracted from *Vitex negundo* Linn leaves has been reported to possess antioxidant and antimelanogenic activities [20]. By contrast, it has been demonstrated that the lotus flower essential oil stimulates melanin synthesis and tyrosinase activity in normal human melanocytes [21]. The *C. cassia* essential oil (CC-EO) has hypouricemic [22] and antifungal activities [23]; however, little information is available regarding the inhibitory action of CC-EO on tyrosinase activity and melanogenesis. Therefore, this study sought to investigate the chemical composition of CC-EO by gas chromatography-mass spectrometry (GC-MS) and examine the tyrosinase inhibitory activity of this EO. To address this inhibitory activity, the effects of CC-EO and its chemical components on the α-MSH-stimulated melanogenesis in B16 melanoma cells were assessed.

## 2. Results and Discussion

### 2.1. The Chemical Composition of CC-EO

The steam-distilled essential oil of CC-EO was analyzed by GC-MS. In total, 16 primary compounds of CC-EO were identified; these compounds, their retention times and their Kovats indices are listed in Table 1. Our results indicated that the two major constituents of CC-EO were *cis*-2-methoxycinnamic acid (43.06%) and cinnamaldehyde (42.37%). A previous study reported that cinnamaldehyde (92.2%) is the most plentiful constituent of the *C. cassia* essential oil [23]. Many factors including harvesting time of the aromatic plant, climatic and agronomic conditions, vegetative cycle stage, different age and segment of the plant, the plant part used, extraction processes and assay methods have been reported to contribute to discrepancies in the observed cinnamaldehyde levels of various *C. cassia* essential oils [24]. Geng *et al.* described that the identified compounds of *C. cassia* bark essential oil showed high fluctuations in the percentages compositions and the majority compound, *trans*-cinnamaldehyde, percentages varied within 33.95%–76.4% [24].

### 2.2. The Inhibitory Effects of CC-EO and Its Major Constituents on Mushroom Tyrosinase Activity

Several investigations have demonstrated that cinnamaldehyde and 2-methoxycinnamic acid possessed potent tyrosinase inhibitory activity [16,25,26]. Therefore, we hypothesized that CC-EO, which contains *cis*-2-methoxycinnamic acid and cinnamaldehyde, might have potent anti-tyrosinase and anti-melanogenic activities. The mushroom tyrosinase has been widely used as a target enzyme for screening and characterizing potential inhibitors of tyrosinase and/or melanogenesis. Next, the effects of CC-EO, cinnamaldehyde and 2-methoxycinnamic acid were examined. As shown in Table 2, the activity of mushroom tyrosinase were significantly inhibited by both CC-EO and cinnamaldehyde in a dose-dependent manner, displaying half-inhibition concentration (IC_50_) values of 6.16 ± 0.04 mg/mL and 4.04 ± 0.08 mg/mL, respectively. In contrast, the other major constituent of CC-EO, *cis*-2-methoxycinnamic acid, could not inhibit the mushroom tyrosinase activity (data not shown). Conversely, a previous study demonstrates that 2-methoxycinnamic acid isolated from *Pulsatilla cernua* root is a potent tyrosinase inhibitor with an IC_50_ value of 0.34 mM [25]. The reason for the discrepant findings in tyrosinase inhibitory property of 2-methoxycinnamic acid remains to be determined, but may relate to differences in the cell culture conditions, assay methods or compound purities. Moreover, the biological activities of plant extracts and essential oils are related to their bioactive components, which may be affected by seasons, geographical origins, harvest times, agronomic practices, and extraction methods [27].

### 2.3. The Effects of CC-EO and *Trans*-Cinnamaldehyde on the Cell Viability, Melanin Content and Tyrosinase in *α*-MSH-Stimulated B16 Cells

**Figure 1:**
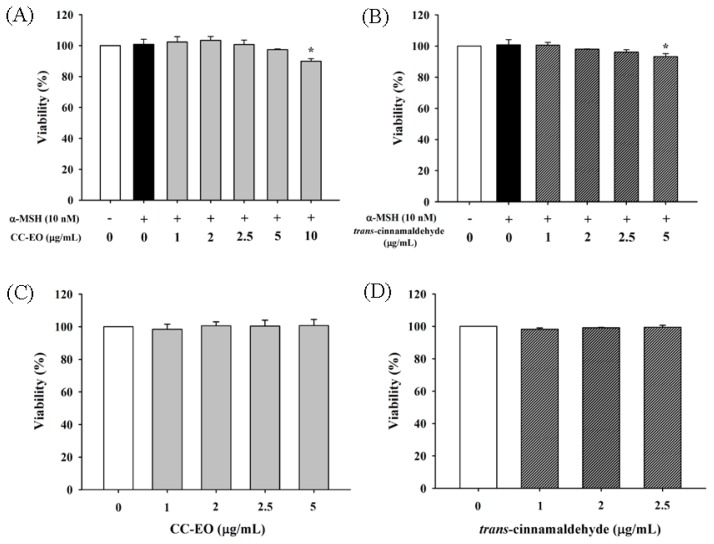
The effects of CC-EO and *trans*-cinnamaldehyde on the cell viability of α-MSH-treated B16 cells (**A**,**B**) and WS-1 cells (**C**,**D**), respectively. The data are represented as the means ± S.D. of three independent experiments. * indicates a significant difference (*p* < 0.05) compared with the untreated group.

To evaluate the effects of CC-EO and cinnamaldehyde on α-MSH-stimulated (10 nM) B16 melanoma cells and to determine the optimal concentrations for the following analyses, MTT assay was used to assess the effects of CC-EO and cinnamaldehyde on cell viability. As shown in Figure 1A,B, CC-EO and cinnamaldehyde have no cytotoxicity to α-MSH-stimulated B16 melanoma cells at concentrations up to 5 μg/mL and 2.5 μg/mL, respectively. In addition, we demonstrated that CC-EO and cinnamaldehyde, at the tested concentrations of 1.0–5.0 μg/mL and 1.0–2.5 μg/mL, respectively, had no significant toxic effects on human fetal skin fibroblast WS1 cell line (Figure 1C,D). Hence, these doses were chosen for further experiments.

**Figure 2:**
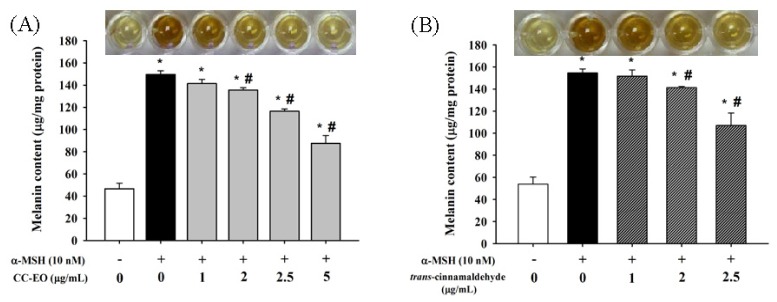
The effects of CC-EO (**A**) and *trans*-cinnamaldehyde (**B**) on melanin content in α-MSH-treated B16 cells. The data are represented as the means ± S.D. of three independent experiments. * indicates a significant difference (*p* < 0.05) compared with the untreated group; ^#^ indicates a significant difference (*p* < 0.05) compared with the α-MSH-treated group.

To evaluate the effects of CC-EO and cinnamaldehyde on melanin production and tyrosinase activity, the B16 melanoma cells were exposed to α-MSH (10 nM) for 72 h in the presence of either CC-EO at 1.0, 2.0, 2.5, or 5.0 μg/mL or cinnamaldehyde at 1.0, 2.0, or 2.5 μg/mL. Following this exposure, the melanin content and tyrosinase activity were measured. The amounts of melanin were sharply increased by approximately three-fold upon exposure to α-MSH for 72 h (Figure 2). However, co-treatments with CC-EO (Figure 2A) or cinnamaldehyde (Figure 2B) significantly inhibited α-MSH-stimulated melanin production in a dose-dependent manner. In particular, compared with the α-MSH alone treatment, the co-administration of 5.0 μg/mL CC-EO (Figure 2A) and 2.5 μg/mL cinnamaldehyde (Figure 2B) significantly reduced melanin production by 42% and 31%, respectively.

**Figure 3:**
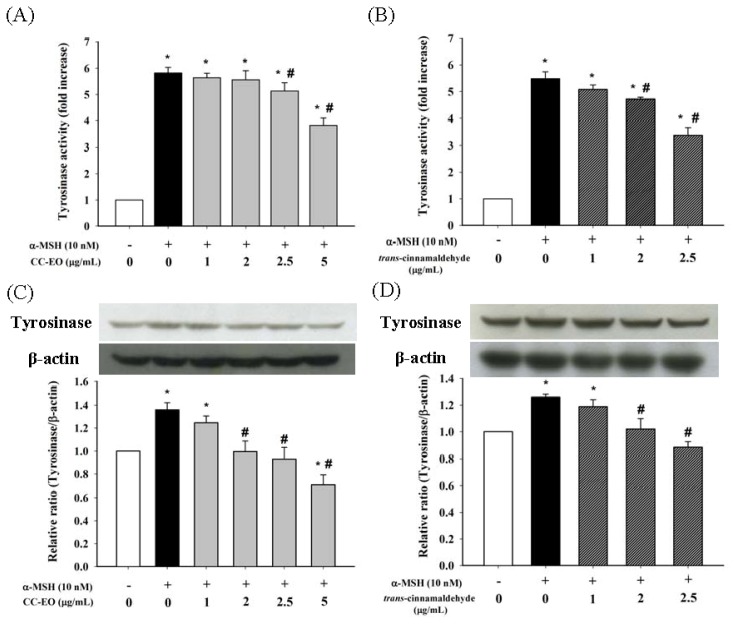
The effects of CC-EO and *trans*-cinnamaldehyde on cellular tyrosinase activity (**A**,**B**) and tyrosinase expression (**C**,**D**) in α-MSH-treated B16 cells, respectively. The data are represented as the means ± S.D. of three independent experiments. * indicates a significant difference (*p* < 0.05) compared with the untreated group; ^#^ indicates a significant difference (*p* < 0.05) compared with the α-MSH-treated group.

Melanogenesis is regulated by the activity of tyrosinase, which is a rate-limiting enzyme in melanin biosynthesis. Thus, the effects of CC-EO or cinnamaldehyde on tyrosinase activity in α-MSH-stimulated B16 melanoma cells were examined. As shown in Figure 3A,B, approximately a five- to six-fold increase in cellular tyrosinase activity was observed in α-MSH-stimulated cells compared with unstimulated control cells. However, treatments with CC-EO and cinnamaldehyde dose-dependent reduced the tyrosinase activity in α-MSH-stimulated B16 melanoma cells. To clarify the further mechanism of tyrosinase inhibition by CC-EO and cinnamaldehyde, the levels of tyrosinase expression in B16 cells were examined by Western blot analysis. The levels of tyrosinase protein were 1.3-fold higher in the α-MSH-stimulated B16 melanoma cells than in the unstimulated control cells (Figure 3C,D). Treatments with both CC-EO and cinnamaldehyde suppressed the α-MSH-induced expression of tyrosinase in a dose-dependent manner. These results indicated that CC-EO and its major constituent, cinnamaldehyde, suppressed α-MSH-stimulated melanogenesis in murine B16 melanoma cells through non-cytotoxic mechanisms that involved the inhibition of tyrosinase activity and the down-regulation of tyrosinase expression, consequently leading to decreases of melanin content.

### 2.4. The Effects of CC-EO and *Trans-*Cinnamaldehyde on MDA Production, GSH Levels and Antioxidant Enzyme Activities in *α*-MSH-Stimulated B16 Cells

The oxidation of l-DOPA generates a highly reactive intermediate that is further oxidized to form melanin through a radical-coupling pathway. In fact, melanogenesis has been reported to involve, not only the production of hydrogen peroxide (H_2_O_2_) by means of enzymatic and non-enzymatic reactions, but also the subsequent generation of other reactive oxygen species (ROS), which cause oxidative stress for melanocytes [28,29]. Oxidative stress is considered to be the result of an imbalance between oxidants and antioxidants within the cell. Furthermore, α-MSH-induced melanogenesis was associated with ROS generation [30]. Therefore, antioxidants, tyrosinase inhibitors, or ROS scavengers may down-regulate melanogenesis. Many previous studies have reported that various antioxidants, including gallic acid [8], vanillin [9], curcumin [11], and ascorbic acid [31], may produce anti-melanogenic effects. Previous studies have demonstrated that both extracts and essential oils from *C. cassia* exhibit antioxidant activity [32,33]. In addition, the essential oil from the related cinnamon species *C. osmophloeum* and its major constituent, *trans*-cinnamaldehyde, exerted significant *in vivo* antioxidant activities against juglone-induced oxidative stress in *Caenorhabditis elegans* [34]. Therefore, we investigated whether exposure to CC-EO and cinnamaldehyde would reduce the oxidative stress in α-MSH-stimulated cells.

**Figure 4:**
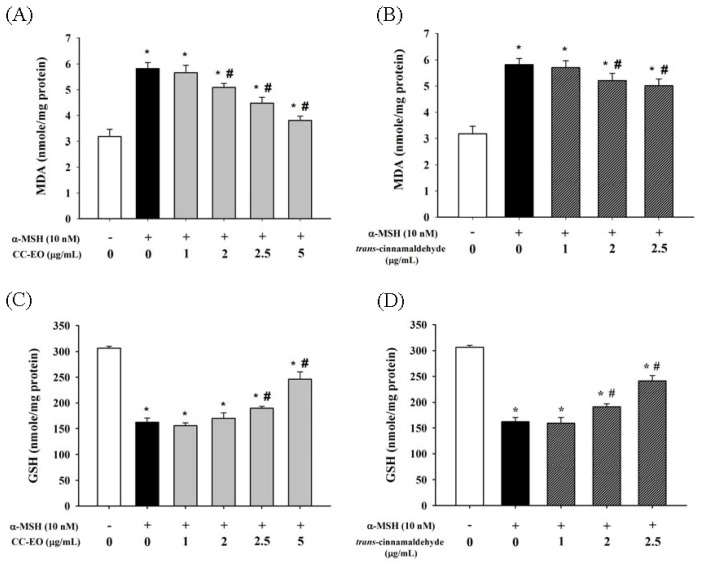
The effects of CC-EO and *trans*-cinnamaldehyde on lipid peroxidation (**A**,**B**) and GSH levels (**C**,**D**) in α-MSH-treated B16 cells, respectively. The data are represented as the means ± S.D. of three independent experiments. * indicates a significant difference (*p* < 0.05) compared with the untreated group; ^#^ indicates a significant difference (*p* < 0.05) compared with α-MSH-treated group.

First, the lipid peroxidation through the production of MDA was measured to determine the cellular oxidative stress that was induced by α-MSH. Compared with unstimulated control cells, cells that were exposed to α-MSH exhibited a 1.8-fold increase in cellular MDA formation. However, treatments with CC-EO and cinnamaldehyde resulted in a dose-dependent attenuation of the α-MSH-stimulated formation of cellular oxidants (Figure 4A,B). The GSH level is important for maintaining cellular redox status and plays an important role in the inhibition of melanogenesis [35]. Our data indicated that compared with unstimulated control cells, stimulation by α-MSH caused a 47% decrease in GSH levels (*p* < 0.05); however, a dose-dependent recovery of α-MSH-reduced GSH levels was observed in the presence of CC-EO and cinnamaldehyde (Figure 4C,D).

**Figure 5:**
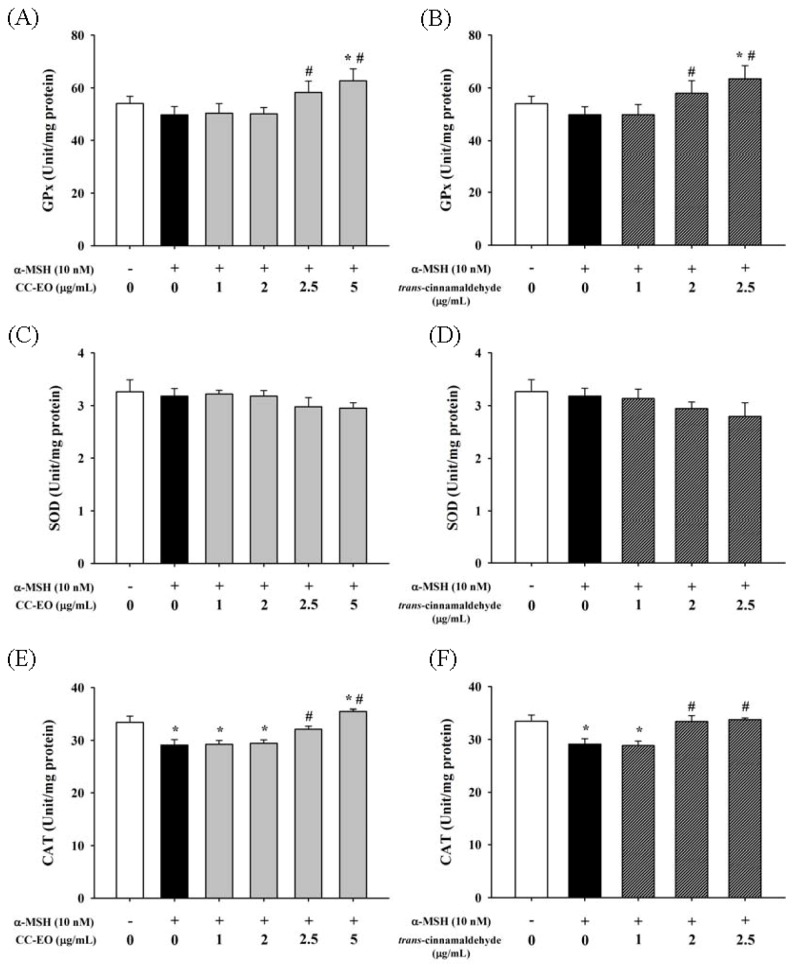
The effects of CC-EO and *trans*-cinnamaldehyde on GPx (**A**,**B**); SOD (**C**,**D**) and CAT (**E**,**F**) activities in α-MSH-treated B16 cells, respectively. The data are represented as the means ± S.D. of three independent experiments. * indicates a significant difference (*p* < 0.05) compared with the untreated group; ^#^ indicates a significant difference (*p* < 0.05) compared with the α-MSH-treated group.

Various antioxidant enzymes, such as GPx, SOD and CAT, play important roles in maintaining the redox homeostasis within cells. The exposure of cells to α-MSH caused a significant decrease in CAT activity compared with unstimulated control cells; however, α-MSH-stimulation did not influence the GPx and SOD activities in cells (Figure 5). Co-treatments with 2.5 or 5.0 μg/mL of CC-EO or with 2.0 or 2.5 μg/mL of cinnamaldehyde significantly increased the activities of both GPx and CAT in α-MSH-stimulated cells. Furthermore, the activities of GPx and CAT in 5.0 μg/mL CC-EO and α-MSH -cotreated cells were significantly higher than the activities of these enzymes in either α-MSH-stimulated cells or unstimulated control cells.

Our data demonstrated that the depletion of GSH content and CAT activity and the aggravation of oxidative stress might be involved in the α-MSH-induced melanogenesis of B16 melanoma cells. CC-EO and its major constituent, cinnamaldehyde, repressed the oxidative stress and lipid peroxidation of α-MSH-stimulated cells because this treatment increased both the levels of GSH and the activities of CAT and GPx within these cells, thereby reducing oxidant generation. Therefore, we can propose that CC-EO and its major component, cinnamaldehyde, possess potent anti-tyrosinase and anti-melanogenic activities that are coupled with antioxidant properties in this study. The pro-opiomelanocortin peptides such as α-MSH and ACTH play an important role in regulation of melanogenesis due to the up-regulation of the cAMP pathway which was involved activation of protein kinase A, cAMP response element binding protein (CREB) transcription factor, microphthalmia associated transcription factor (MITF), and melanogenic-related enzymes such as tyrosinase and TRPs [36,37]. Recent studies had indicated that the up-regulation of cAMP also activates MAPK (ERK1/2) in B16 melanoma cells [38,39] and ERKs signaling led to decreased tyrosinase expression and melanogenesis via MITF phosphorylation and degradation [40,41]. Conversely, p38 MAPK, one member of MAPK family, was found to be activated in B16 cells by α-MSH [42] and implicated in melanogenesis via stimulation MITF expression and promotion tyrosinase gene expression [43]. Moreover, MAPK had been shown to play a key role in the regulation of cellular response to oxidative stress [44], and ERKs and p38 MAPK were shown to be involved in stress-induced melanogenesis [45,46]. Since CC-EO regulated the cellular oxidative stress induced by α-MSH in our study, the relationship between anti-melanogenesis effect and the MAPK regulation of CC-EO should be investigated in the future.

## 3. Experimental Section

### 3.1. Essential Oils

The essential oil, which was obtained by steam distillation from the stem bark of *C. cassia* Presl, was purchased from Yangsen Biotech, Inc. (Taipei, Taiwan). The extraction procedure was accomplished in accordance with the methods of a previously published study, with slight modifications [47]. Briefly, *C. cassia* stem bark was placed into a vessel and extracted by distillation for 4 h. The vapors were cooled by a closed cooling system, and the resulting liquid was collected in a container. The oils floated towards the top of the distilled liquid, whereas the water settled into the lower phase of this liquid; thus, the essential oils were obtained by simply removing the upper phase of the liquid, which contained the desired oils.

### 3.2. Chemicals

The compounds of *trans*-cinnamaldehyde, mushroom tyrosinase (EC 1.14.18.1; T7755), α-melanocyte stimulating hormone (α-MSH), dimethyl sulfoxide (DMSO), monobromobimane (MbBr) and phenylmethylsulfonyl fluoride (PMSF) were purchased from Sigma-Aldrich Chemicals Co. (St. Louis, MO, USA). The l-3,4-dihydroxyphenylalanine (l-DOPA) that was used in this study was purchased from Merck (Darmstadt, Germany). Dulbecco’s modified eagle medium (DMEM), minimum essential medium (MEM), fetal bovine serum (FBS), l-glutamine, penicillin-streptomycin, and trypsin-ethylenediaminetetraacetic acid were purchased from Invitrogen Life Technologies (Carlsbad, CA, USA). All of the other chemicals that were used were of at least reagent-grade quality.

### 3.3. GC-MS Analysis

The *C. cassia* essential oil was analyzed by GC-MS to identify its constituents. The GC-MS (GCMS-QP2010 Plus; Shimadzu, Tokyo, Japan) was equipped with a Forte ID-BPX5 column (30.0 m × 0.25 mm *i.d.*, 0.25 μm film thickness; SGE, Australia), and the injector temperature was maintained at 250 °C. Helium (He) was used as the carrier gas at a flow rate of 1 mL/min, and the split ratio was set at 1:100. The oven temperature was initially held at 50 °C for 5 min and then programmed to increase by 5 °C/min to 150 °C; from this point, the temperature was ultimately raised to 300 °C at a rate of 10 °C/min. The mass spectrometry conditions were as follows: a scan range of 40–350 amu, an ion source temperature of 230 °C and an interface temperature of 250 °C. The essential oil constituents were identified by comparing the retention times and retention indices of the chromatographic peaks with the retention times and retention indices of authentic reference standards that were assessed using the same conditions. An assessment of the peak enrichment on co-injection with authentic reference compounds was also conducted. The 2005 version of the National Institute of Standards and Technology (NIST) MS spectral database was used to perform a comparison of the MS fragmentation pattern with the fragmentation patterns of pure compounds and a mass spectrum database search.

### 3.4. Enzymatic Assay of Mushroom Tyrosinase

The tyrosinase inhibitory activities of the CC-EO and its major constituents were determined by the tyrosinase-dependent l-3,4-dihydroxyphenylalanine (l-DOPA) oxidation assay according to a slight modification of the method of Kubo and Kinst-Hori [18]. The substrate solution (0.84 mL of 0.89 mM l-DOPA in 16 mM sodium phosphate buffer, pH 6.8) was incubated at 25 °C for 10 min. Following incubation, 0.03 mL of each sample solution and 0.03 mL of mushroom tyrosinase (1000 units/mL; T7755, Sigma, one unit = Δ*OD*_280_ of 0.001 per min at pH 6.5 at 25 °C in 3 mL reaction mixture containing l-tyrosine) were added. The assay mixture in a total volume of 0.9 mL was immediately monitored for the formation of dopachrome by measuring the linear increase in optical density at 475 nm. The inhibitory percentage of tyrosinase was calculated as follows: % inhibition = (1 − *B*/*A*) × 100, where *A* = Δ*OD*_475_/min without tested sample and *B* = Δ*OD*_475_/min with tested sample. The 50% inhibition (IC_50_) of tyrosinase activity was calculated as the concentrations of the tested sample that inhibited 50% of tyrosinase activity under experimental conditions.

### 3.5. Cell Cultures

The B16 murine melanoma cell line and WS1 human fetal skin fibroblast cell line were purchased from the Bioresource Collection and Research Center (BCRC; Hsinchu, Taiwan). The B16 cells and WS1 cells were cultured in DMEM and MEM, respectively; both types of culture medium were supplemented with 10% FBS, 2 mM glutamine, 100 mg/mL streptomycin, and 100 U/mL penicillin. The cells were maintained in a humidified 5% CO_2_ incubator at 37 °C and were sub-cultured every 3–4 days to maintain logarithmic growth.

### 3.6. Cell Viability

The B16 cells were seeded in a 96-well plate at a density of 5 × 10^3^ cells/well. After the incubation of this plate for 24 h, different concentrations of samples and 10 nM α-MSH were added to each well once; the plate was then incubated for an additional 72 h. The WS1 cells were seeded in a 48-well plate at a density of 4 × 10^4^ cells/well. After the incubation of this plate for 24 h, different concentrations of samples were added to each well, and the plate was then incubated for an additional 24 h. The cell viability was determined by the improved MTT assay [48].

### 3.7. The Assay of Melanin Content

The method of Thanigaimalai *et al.* [49] was used to determine the melanin content of the B16 cells. These cells were incubated with various concentrations of CC-EO (1.0, 2.0, 2.5 or 5.0 μg/mL) or *trans*-cinnamaldehyde (1.0, 2.0 or 2.5 μg/mL) and were subsequently co-treated with 10 nM α-MSH once for 72 h. Following this treatment, the cells were dissolved in 1 M NaOH/10% DMSO solution and incubated at 90 °C to solubilize the melanin. The total melanin in each cell suspension was determined by recording the absorbance of each suspension at 405 nm. The melanin content was calculated by interpolating the results onto a standard curve that was generated by the absorbance of known concentrations of synthetic melanin and correcting for the total amounts of protein that are present in the supernatants of cell lysates.

### 3.8. The Assay of Cellular Tyrosinase Activity

Cellular tyrosinase activity was assayed in terms of DOPA oxidase activity, using the method of Seo *et al.* [50]. The B16 cells were incubated with various concentrations of CC-EO (1.0, 2.0, 2.5 or 5.0 μg/mL) or *trans*-cinnamaldehyde (1.0, 2.0 or 2.5 μg/mL) and were subsequently co-treated with 10 nM α-MSH once, for 72 h. At the endpoint of this treatment, the cells were sonicated with phosphate buffer (pH 6.8) containing 1 mM PMSF. The lysates were clarified by 10 min of centrifugation at 10,000 × *g*. Following the protein quantification of each lysate and protein concentration adjustments with lysis buffer, 300 μL of each lysate was mixed with 700 μL of 5 mM l-DOPA; this mixture was incubated for 1 h at 37 °C, and the absorbance was then measured spectrophotometrically at 475 nm.

### 3.9. Western Blot Analysis

The B16 cells were incubated with various concentrations of CC-EO (1.0, 2.0, 2.5 or 5.0 μg/mL) or *trans*-cinnamaldehyde (1.0, 2.0 or 2.5 μg/mL) and subsequently co-treated with 10 nM α-MSH once for 72 h. Whole cell extracts were prepared with PRO-PREP^TM^ protein extraction solution (iNtrRON Biotechnology, Gyeonggi-do, Korea). The protein concentration was determined using the BCA protein assay kit (Pierce, Rockford, IL, USA). The aliquots of cell lysates were separated by electrophoresis on SDS-polyacrylamide gels and transferred to PVDF membranes. The membranes were blocked with 5% dried milk in phosphate buffer saline containing 0.1% Tween 20 and were then probed with appropriate antibodies. The anti-tyrosinase and anti-β-actin antibodies were used at 1:1000 and 1:5000, respectively (Abcam, MA, USA). The horseradish peroxidase-conjugated goat anti-rabbit and horseradish peroxidase-conjugated goat anti-mouse IgGs were used at 1:10000 (Millipore, Germany). The antibody complexes were detected by chemiluminescence (ECL; Millipore, Germany). The protein levels were quantified by UVIpro Platinum 1.1 (Version 12.9; UVItec, UK).

### 3.10. The Measurement of the Lipid Peroxide and Glutathione (GSH) Levels and the Glutathione Peroxidase (GPx), Superoxide Dismutase (SOD), and Catalase (CAT) Activities

B16 cells were incubated with various concentrations of CC-EO (1.0, 2.0, 2.5, or 5.0 μg/mL) or *trans*-cinnamaldehyde (1.0, 2.0 or 2.5 μg/mL) and subsequently co-treated with 10 nM α-MSH once, for 72 h. At the endpoint of this treatment, the cells were harvested and sonicated with phosphate buffer (pH 6.8) containing 1 mM PMSF to obtain cell homogenates. The thiobarbituric acid-reactive substances (TBARS) method was used to estimate cellular malondialdehyde (MDA) levels with a spectrophotometer by measuring the absorbance at 535 nm [51]. The cellular GSH was reduced with a dithiothreitol/phosphate solution and derivatized with MbBr prior to the conduction of HPLC analysis [52].

With respect to GPx activity, one unit of GPx activity was defined as the amount of the enzyme that oxidized 1 nM of NADPH per min, as measured by the absorbance readings that were obtained at 340 nm [53]. Superoxide dismutase (SOD) activity was determined spectrophotometrically at 325 nm by determining on the SOD-mediated decrease in the rate of pyrogallol autoxidation under alkaline conditions [54]. One unit of SOD activity was defined as the amount of the enzyme that inhibited the rate of pyrogallol oxidation. Catalase (CAT) activity was analyzed by measuring the decrease in absorbance of H_2_O_2_ at 240 nm. One unit of CAT activity was defined as the amount of the enzyme that decomposed 1.0 μM of H_2_O_2_ per min [55]. The specific activities of SOD, CAT, and GPx are expressed in terms of units/mg protein.

### 3.11. Statistical Analysis

All tests were carried out independently in triplicate (*n* = 3). The data were expressed as the mean ± standard derivation (S.D.). One-way analysis of variance (ANOVA) was used to determine the significant differences between the groups followed by a Scheffe test for multiple comparisons. A *p*-value < 0.05 was considered to be statistically significant. All analyses were performed using SPSS version16.0 (SPSS Inc., Chicago, IL, USA).

## 4. Conclusions

To the best of our knowledge, this study is the first report that states that CC-EO and its major constituent, cinnamaldehyde, could effectively inhibit melanin production in B16 melanoma cells. Our observations indicated that CC-EO and cinnamaldehyde inhibited α-MSH-induced melanogenesis through the tyrosinase inactivation and the simultaneous suppression of oxidative stress in B16 melanoma cells. Both CC-EO and cinnamaldehyde are generally recognized as safe (GRAS), although cinnamaldehyde is a potent skin sensitizer. Therefore, CC-EO and cinnamaldehyde could potentially be employed as effective skin-whitening agents and as antioxidants for the future development of complementary and alternative medicine-based aromatherapy.

## Figures and Tables

**Table 1:** The gas chromatography-mass spectrometry (GC/MS) analysis of CC-EO.

Compounds	MF^a^	Rt^b^	KI^c^	Area %
Benzaldehyde	C_7_H_6_O	7.58	982	0.42
2,2,4,6,6-Pentamethylheptane	C_12_H_26_	7.84	983	0.21
2,5,9-Trimethyldecane	C_13_H_28_	9.10	1121	0.49
2,5-Dimethylundecane	C_13_H_28_	9.49	1136	0.33
Phenylethyl alcohol	C_8_H_10_O	11.79	1175	0.29
Cinnamaldehyde	C_9_H_8_O	16.57	1189	42.37
3,4-Dimethoxyphenethyl alcohol	C_10_H_14_O_3_	17.78	1514	0.79
Germacrene D	C_15_H_24_	18.97	1515	0.32
*cis*-2-Methoxycinnamic acid	C_10_H_10_O_3_	19.69	1546	43.06
Cinnamyl acetate	C_11_H_12_O_2_	20.88	1589	1.83
Coumarin	C_9_H_6_O_2_	20.99	1623	1.25
*o*-Methoxycinnamaldehyde	C_10_H_10_O_2_	22.70	1745	5.11
*trans*-Caryophyllene	C_15_H_24_	22.95	1832	0.43
1,2-Dimethoxy-4-(3-methoxy-1-propenyl) benzene	C_12_H_16_O_3_	24.11	1946	2.05
2-Ethyl-5-propylphenol	C_11_H_16_O	24.78	1993	0.21
β-Phenethyl cinnamate	C_17_H_16_O_2_	32.01	2041	0.16

a Molecular formula;

b Retention time (min);

c Kovats index.

**Table 2:** The tyrosinase inhibitory activities of CC-EO and *trans*-cinnamaldehyde.

Concentration (mg/mL)	Inhibition of tyrosinase activity (%)	

	CC-EO	*trans*-cinnamaldehyde
0.00	0.00 ± 0.00	0.00 ± 0.00
0.78	16.70 ± 0.41	16.20 ± 0.58
1.56	27.26 ± 0.58	29.17 ± 0.84
3.13	38.28 ± 0.07	45.90 ± 0.89
6.25	50.83 ± 0.35	63.11 ± 0.54
12.50	62.41 ± 0.34	77.03 ± 0.46
25.00	72.12 ± 0.34	85.62 ± 0.20
50.00	79.83 ± 0.07	91.18 ± 0.18

Values are displayed as means ± S.D. (*n* = 3).
